# Drought Stress Impacts on Plants and Different Approaches to Alleviate Its Adverse Effects

**DOI:** 10.3390/plants10020259

**Published:** 2021-01-28

**Authors:** Mahmoud F. Seleiman, Nasser Al-Suhaibani, Nawab Ali, Mohammad Akmal, Majed Alotaibi, Yahya Refay, Turgay Dindaroglu, Hafiz Haleem Abdul-Wajid, Martin Leonardo Battaglia

**Affiliations:** 1 Plant Production Department, College of Food and Agriculture Sciences, King Saud University, P.O. Box 2460, Riyadh 11451, Saudi Arabia; nsuhaib@ksu.edu.sa (N.A.-S.); malotaibia@ksu.edu.sa (M.A.); refay@ksu.edu.sa (Y.R.); 438106@ksu.edu.sa (H.H.A.-W.); 2 Department of Crop Sciences, Faculty of Agriculture, Menoufia University, Shibin El-Kom 32514, Egypt; 3 Department of Agronomy, University of Agriculture Peshawar, Peshawar 25130, Pakistan; nawab@aup.edu.pk (N.A.); akmal@aup.edu.pk (M.A.); 4 Livestock research and development station, Surezai Peshawar, Peshawar 25000, Pakistan; 5 Department of Forest Engineering, Faculty of Forestry, Kahramanmaras Sutcu Imam University, Kahramanmaras 46100, Turkey; turgaydindaroglu@ksu.edu.tr; 6 Department of Animal Sciences, Cornell University, Ithaca, NY 14850, USA

**Keywords:** drought stress, plants, mitigation, abiotic stress

## Abstract

Drought stress, being the inevitable factor that exists in various environments without recognizing borders and no clear warning thereby hampering plant biomass production, quality, and energy. It is the key important environmental stress that occurs due to temperature dynamics, light intensity, and low rainfall. Despite this, its cumulative, not obvious impact and multidimensional nature severely affects the plant morphological, physiological, biochemical and molecular attributes with adverse impact on photosynthetic capacity. Coping with water scarcity, plants evolve various complex resistance and adaptation mechanisms including physiological and biochemical responses, which differ with species level. The sophisticated adaptation mechanisms and regularity network that improves the water stress tolerance and adaptation in plants are briefly discussed. Growth pattern and structural dynamics, reduction in transpiration loss through altering stomatal conductance and distribution, leaf rolling, root to shoot ratio dynamics, root length increment, accumulation of compatible solutes, enhancement in transpiration efficiency, osmotic and hormonal regulation, and delayed senescence are the strategies that are adopted by plants under water deficit. Approaches for drought stress alleviations are breeding strategies, molecular and genomics perspectives with special emphasis on the omics technology alteration i.e., metabolomics, proteomics, genomics, transcriptomics, glyomics and phenomics that improve the stress tolerance in plants. For drought stress induction, seed priming, growth hormones, osmoprotectants, silicon (Si), selenium (Se) and potassium application are worth using under drought stress conditions in plants. In addition, drought adaptation through microbes, hydrogel, nanoparticles applications and metabolic engineering techniques that regulate the antioxidant enzymes activity for adaptation to drought stress in plants, enhancing plant tolerance through maintenance in cell homeostasis and ameliorates the adverse effects of water stress are of great potential in agriculture.

## 1. Introduction

Plants are exposed to various environmental stresses during growth and development under natural and agricultural conditions. Among these, drought is one the most severe environmental stresses affecting plant productivity. About 80–95% of the fresh biomass of the plant body is comprised of water, which plays a vital role in various physiological processes including many aspects of plant growth, development, and metabolism [1,2]. As a result, some consider drought as the main environmental stress for different plants, particularly in drought prone areas [3,4], the single most critical threat to world food security in the future and the catalyst of important famines in the past [5]. The effects of drought in agriculture are aggravated due to the depletion of water resources and the increased food demand from an alarming world population growth [6]. The unpredictable nature of the drought is dependent upon various factors such as uneven and undependable distribution of rainfall, evapotranspiration, and water holding capacity around the rhizosphere [7,8]. Moreover, in some cases plants are unable to uptake water from the soil, even though enough moisture is present in the root zone [9], a phenomenon known as physiological drought or pseudo-drought [10].

**Figure 1:**
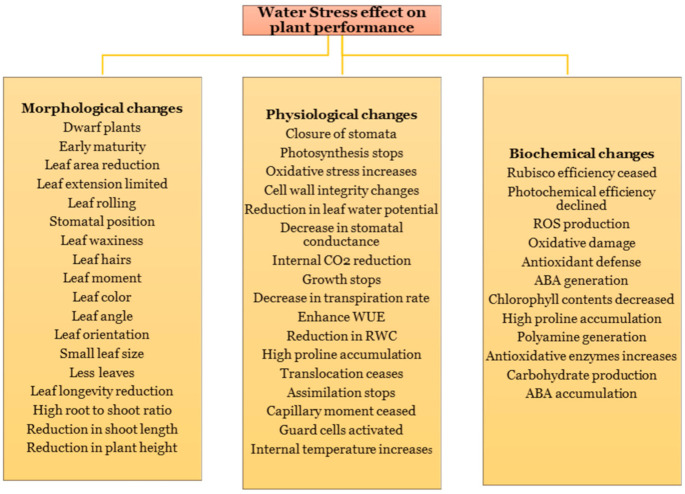
Morphological, physiological and biochemical dynamics of plants affected by water stress.

Different molecular, biochemical, physiological, morphological and ecological traits and processes (Figure 1) of the plants are impaired under drought stress conditions [11]. Plant yield and quality are adversely affected in water deficit environments [12]. Growth stages, age, plant species and drought severity and duration are the key factors that influence the plant responses to drought [13]. The resistance mechanism to drought, in turn, varies among plant species. Plants, therefore, have the ability to reduce their resource utilization and adjust their growth to cope against adverse environmental conditions like drought [14,15]. Various networks at the molecular level, such as those involved in signal transduction, are responsible for enhancing these responses against drought stress [16,17]. The stomatal regulation of plants through enhanced ion transport, transcription factor activities and abscisic acid (ABA) signaling are also involved in the molecular mechanisms of plant response to drought stress [18,19].

Under certain changing circumstances, there is a need to improve the drought tolerance of the plant. For enhancement of water-use efficiency, when physical adaptation of roots and leaves are not enough to cope with certain drought molecular signals including the gene coding regularity protein that expresses many other genes and signals through crosstalk according to different regulatory mechanisms [20,21]. To meet future food demand, fostering more work on drought-tolerant plants and the use of economical and beneficial agriculture practices will be of paramount importance [22,23].

## 2. Causes of Drought Stress in Plants

Global climate change is expected to accelerate in the future because of the continuous rising of air temperature and atmospheric CO_2_ levels that ultimately alters the rainfall patterns and its distribution [24,25]. Although deficient water input from rainfall is usually the main driver for drought stress, the loss of water from soils through evaporation, which is driven by high temperature events, high light intensity and dry wind, can further aggravate an existing drought stress event [26]. Global climate change typically results in prevalent drought stress conditions over vast areas at a global scale. Alongside drought, salinity stress is also considered a primary cause of water deficit in plants [27,28,29]. Certain factors responsible for drought stress are briefly highlighted.

### 2.1. Global Warming

Some of the consequences derived from climate change could be beneficial for agricultural productivity. For example, higher rates of photosynthesis have been reported under elevated CO_2_, hence its presence in the atmosphere in elevated concentrations could enhance grain yields in the future [30]. However, in most cases, climate change has detrimental consequences both in natural and agricultural ecosystems. Increases in air temperatures can result in the melting of glaciers and potential flooding of agricultural lands with low or null slope [31]. Additionally, the loss of glaciers is causing the shrinkage of water reservoirs which limits the water availability to crops, a trend that is increasing with time. In fact, in various rain-fed agricultural areas around the world, the annual accumulated precipitation has decreased because of global warming [32]. Loss of water due to global warming is not only occurring in the soil, but also at the plant level. Internal water in plants up to great extent are lost to the atmosphere driven by the increased temperatures resulting from global warming, a phenomenon that further exacerbates the already existing water deficit problems in various agricultural systems around the world [33]. If expected increases in air temperature around 2 °C greater than present levels occur by the end of this century, approximately one fifth of the world population will be affected by severe water deficit [34].

### 2.2. Rainfall Anomalies

More stress is expected in areas where crop production is solely dependent on rainfall compared to areas that are being irrigated through canals, rivers and the water channel [35]. Thus, in rain-fed areas drought episodes are strongly correlated with the rainfall distribution across the year and high chances of water stress are observed in some years over a certain period of time [35]. Industrialization, deforestation and urbanization are the prominent anthropogenic activities that affect rainfall patterns, and thus water availability to plants, through its influence in climate change [36]. In Pakistan, erratic and more frequent rainfall occurs in early spring and winter, while more frequently drier and hotter seasons take place due to less and/or no rainfall in early fall and summer seasons. In summer in particular, the combination of greater atmospheric water demand for the plants, higher evaporation and transpiration rates, and less rainfall availability associated with this season amplifies the detrimental effects of drought stress in plant growth and development. However, rainfall distribution and intensity within and across the years play a prominent role in both the management of the water resources for plants and the occurrence of drought stresses in most cases [37,38].

### 2.3. Shifts in Monsoon Patterns

During the summer season, the monsoon system is considered as a source of rainfall in various areas of the world. Its occurrence is interlinked with temperature being the driving force [39]. It is expected that in rain-fed areas the amount of summer precipitations will decrease by 70% by the beginning of the XXII century if the prevailing situations continue [40]. According to estimations, high rainfall is expected due to linear increment in CO_2_ concentration in atmosphere that will affect crop production adversely and will lead to massive floods and massive economic losses in the agriculture sector of densely populated countries [41,42]. Under such circumstances, monsoon rainfall variability is and will continue to affect the moisture level of the rhizosphere, thereby affecting plant productivity in particular areas of the world through dynamics in rainfall intensity, occurrence and duration. Remarkably, two thirds of the world population are currently facing food insecurity due to extreme variation between dry and wet seasonal rainfalls as a result of changes in the monsoon shifts [43]. Added to the intrinsically random and unpredicted nature of the rainfall patterns, and due to recent climate changes, shortening or extendibility of the rainy season may exacerbate present and future scenarios with both water deficit and/or water excess problems in some climatic zones [37]. Being agricultural, crop production practices need to be adopted accordingly during the monsoon behavior and shifted to sustainable crop production. Proper management and crop planning are two strategies to cope with quantitative shifts going from deficient to excessive and, vice versa, to monsoon patterns.

## 3. Effect of Drought Stress on Plants

Depending on the dynamics in the environmental conditions, plants could face various stresses that may severely affect their growth and development [44,45]. Certain metabolic changes and gene expressions occur to enable the plants to survive under these circumstances [27,46]. Grain quality and yield could be greatly affected by drought stress, known as the most limiting stress in agriculture. Thus, investigating the plants’ ability to cope with water limitation is of great value and should continue to receive attention in the near future, especially in arid and semi-arid environments [47]. Currently, major staple crops are being intensively studied to identify the drought-responsive mechanisms to harvest maximum grain yields and quality, but future work should focus on the combined effect of both heat and drought stress impacts at the reproductive stages of main grain crops [48].

The optimal level of water availability is necessary for plants growth and development, fluctuation in soil moisture beyond optimal can affect grain yield and quality. On the other hand, less than optimal water availability in the rhizosphere hampers the plant growth, thereby inhibiting the plant nutrient uptake [49]. The latter has recently been responsible of huge reductions in the production of grain crops, and is only expected to become more severe due to global warming and variability in climate [50,51].

Water scarcity outbreaks are due to the occurrence of less or the absence of rainfall resulting in low soil moisture content and low water potential in aerial parts of the plant such as leaves and stems [52]. When this occurs, the rate of loss of water through transpiration from leaves surpasses the water uptake rate through roots in dry environments [53]. The roots strive to uptake more water through their expansion and this ultimately adapts plants to minimize stomatal loss of water when there is a water deficit [54]. Typical drought stress symptoms in plants include leaf rolling, stunning plants, yellowing leaves, leaf scorching, permanent wilting [55]. Moreover, plant response to a given water deficit is strongly dependent on the previous occurrence and intensity of other drought stress events [28,56,57] and the presence of other stresses [58].

**Figure 2:**
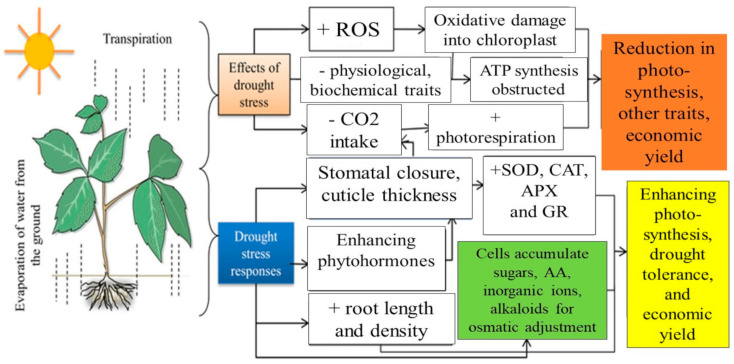
Adverse effects and adaptations of plants to drought stress, modified from Ullah et al. [63]—means reduce; + means increase.

Despite the adverse effects that water deficit has on plant performance, plants have the ability to respond to varying degrees of water deficit (Figure 2). There is a strong correlation between plant growth and water availability as cell enlargement is more affected by water deficits than cell division [59]. Under these conditions, the growth of the plants is inhibited as a result in the reduction of the cell wall extensibility and turgor [60]. When drought conditions are severe, respiration can also decrease, although increments in respiration were observed under mild stress [61]. To cope against the water deficit, the osmotic adjustment of stressed plants is maintained through an increase in sugar content of roots and leaves, and relatively greater growth in roots compared to shoots has been observed in plants subjected to drought stress in the past [62].

Environmental factors including drought duration, intensity and frequency, soil characteristics, growth conditions and stages, and plant species strongly influence the extent and duration of drought-related symptoms in plants [64]. Increases in the rate of leaves senescence and drooping, scorching and limp leaves, leaf rolling and brittleness, closed flowers and flower sagging, etiolation, wilting, turgidity, premature fall, senescence and yellowing of leaves are among the most ubiquitous symptoms of drought stress in plants [65,66]. Although less usual, twig cracks, branch dieback, necrosis, stunted growth, bark crack, shrub canopy and tree thinning represent other symptoms displayed by plants under drought conditions [67]. In some cases, plants may die under extreme drought stress. Whereas water deficiency typically has a profound impact on plant growth and development, water excess also affects plant performance and hampers growth and final yield [68]. When this occurs, excess water stress symptoms are soft fleshy leaves, leaves with rotten patches, fungus affected and moldy plant parts.

## 4. Plant Responses to Drought Stress

**Figure 3:**
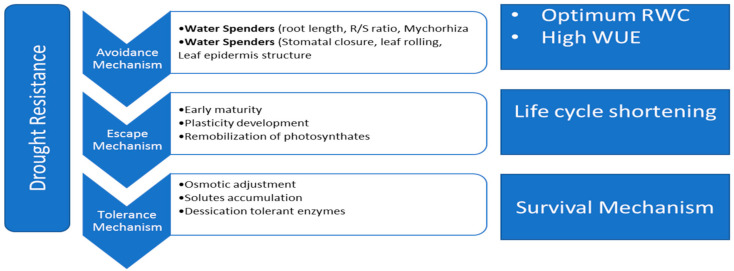
Schematic diagram of drought resistance mechanism in plants. RWC = relative water contents; WUE = water-use efficiency.

Different adaptive mechanisms that make plants more tolerant to the adverse effects of drought stress have been developed through evolution [69]. Stress avoidance, escape and tolerance are the three main survival strategies that plants utilize when exposed to drought stress. Thus, plants responses to drought stress vary from the molecular up to plant level [70]. The mechanisms of plant escape, avoidance and tolerance (Figure 3) against drought stress are discussed in the following sections.

### 4.1. Escape Mechanism

To escape the detrimental effects of drought stress on plant productivity, some plants utilize mechanisms involving rapid plant development and shortening of the life cycle, self-reproduction, and seasonal growth before the beginning of the driest part of the year [71]. Among these mechanisms, early flowering is perhaps the best possible escape adaptive mechanism in plants [72], although this mechanism can imply a considerable reduction in the length of the plant growing period and the final plant productivity in some cases [73].

### 4.2. Avoidance and Tolerance Mechanisms

Under the avoidance strategy, plant water potential is maintained high through a reduction in the stomatal transpiration losses and the increase of water uptake from well-established root systems [74]. In other cases, xeromorphic characteristics such as the presence of hairy leaves and cuticles may help to maintain high water potentials in plant tissues [75]. However, overdevelopment of these structures has a value for the plant in terms of reductions in plant productivity and reduced average size of vegetative and reproductive parts of the plant [76].

On the other hand, an adaptive tolerance mechanism at the photosynthetic machinery level includes reductions in the plant leaf area and limitations in the expansion of new leaves. Similarly, trichomes production on either side of the leaves are exomorphic attributes that allow the plant to tolerate water deficits in dry environments [77]. These structures reduce the leaf temperature by increasing the rate of light reflection in the leaf and also by adding another extra layer of resistance to the water loss. Hence the rate of water loss through leaf transpiration is reduced [78]. However, it is broadly accepted that changes in the root system, including root size, density, length, proliferation, expansion and growth rate, represent the main strategy for drought-tolerant plants to cope against water deficits [79]. Other mechanisms like osmotic adjustment, antioxidant defense mechanism, solute accumulation, metabolic and biochemical dynamics of stomatal closure and increment in root shoot ratio are other common strategies that allow plants to tolerate the adverse effect of drought stress [80].

## 5. Approaches to Alleviate the Adverse Effects of Drought Stress

Use of best management practices related to sowing time, plant population, plant genotype, and soil and nutrient management can help to reduce grain yield losses in field crops subjected to drought stress [81,82]. However, use of transgenic plants with drought-tolerant events is perhaps the drought stress mitigation approach most heavily publicized and the one receiving more attention at present. Several efforts like breeding, molecular and genomic approaches are being undertaken to develop drought-tolerant plants through usual conventional breeding methods [83], with the focus to improve water extraction efficiency, water use efficiency, stomatal conductance, and osmotic adjustments, among others [84]. Other strategies include use of modern and more effective methods of irrigation, good planting practices, mulching, contouring, osmoprotectants and plants inoculations with certain microorganisms that enhance drought tolerance [85].

### 5.1. Selection and Breeding Strategies

Conventional and traditional breeding methods used up to the present were based on the empirical selection of yield [86]. The low heritability, on the one hand, and high genotype and environment interaction on the other, are the main factors defining the quantitative yield trait in major staple crops [87]. Thus, conventional breeding is in practice for yield improvement [88]. Knowledge of plant physiological processes is the prerequisite for selecting quantitative trait loci, locating gene sequences and quantitative trait loci introgression [89]. Due to irregular, undependable and unpredictable response of the drought, screening resistant cultivars is not possible in open conditions, however, it is manageable in sheltered and/or controlled conditions [90]. Conversely, the expression of randomly selected progenies for improved drought stress tolerance in diverse environments is an effective approach known as classical breeding [91]. The cultivars with low transpiration rates and unchanged WUE under non-stress conditions have no effect on final harvest [92]. Scientists are working on the genetic analysis of the root architecture, relative water contents, and osmotic potentials [93]. Focus need to be given to the yield contributing traits which are highly heritable that affects the grain yield under drought conditions but not under optimal conditions based on their feasibility to measure [94]. Nevertheless, they exhibit broad sense heritability for yield in water-limited agriculture systems and have often no interaction with grain yield [95]. When plants are subjected to drought stress, the most important factor that appears first under such circumstances is hampering of WUE which differs for varieties and cultivars [96]. Under these circumstances, plants decrease the stomatal density and leaf size thereby minimizing water loss and maintains the internal water balance [97]. Hence certain genotypes and cultivars, which are drought susceptible and unable to adjust to environmental conditions, resulted in low WUE [98]. Therefore, through a breeding approach, WUE could be enhanced for sustainable crop product in biomass per unit of water utilized [99].

Drought resistance is induced directly or indirectly in the crop species through traits’ genetic variability and thus has the improvement capability through selection in breeding. Marker assisted selection (MAS) and genomic selection (GS) are the two main approaches of genomic assisted breeding. For the prior approach, an initial step is to identify the molecular markers associated with the trait of interest, the prerequisite for selection in breeding programs. However, GS depends on progress of selection models based on genetic markers present on the whole genome and selection of genome estimated breeding values (GEBVs) in breeding populations through phenotyping training population. The MAS is a key part for many crops breeding programs over a few decades, GS being relatively new because it has only recently been applied to crops.

Molecular markers are involved in MAS that map close to quantitative trait loci (QTL) or specific genes that are linked with the particular target trait and could be used identify the individual with desirable alleles [100]. The QTL mapping or genome-wide association approaches are used to select marker trait association through accurate, reliable trait evaluation and dense molecular markers. Through these methods, QTLs for the traits linked with drought resistance are identified in various crops i.e., wheat [101], maize [102], sorghum [103], rice [104], soybean [105], pearl millet [106] and many other crops.

The genomic selection uses all the markers available for a population of GEBVs and GS models are used for selection of elite lines without phenotyping [100]. Contrary to MAS, the knowledge of QTLs is not the prerequisite for GS [107]. However, GS needs higher density marker data than MAS. This is possible through availability of low cost and genome wide marker coverage genotyping approaches [108]. GS is being applied for drought resistance induction breeding in maize by the international maize and wheat improvement center (CIMMYT) [109]. Research efforts through this approach are on course in other crops i.e., sugarcane, legumes and wheat [110,111,112].

### 5.2. Molecular and Genomic Perspective

Biochemical and molecular factors involved in the induction of processes to ameliorate the negative impacts of water stress include transcription, stress responsive genes (Table 1) and abscisic acid [113]. Concurrently to the increased tolerance to drought deficits, breeding programs are also interested to kept other stresses under control through transgenic expression of different stress responsive genes [114,115]. However, the increased expression of these genes is frequently associated with a deceleration in the plant growth rate, this could narrow down its practical use. Thus, the molecular and genetic bases for drought resistance still needs attention to successfully contend with these circumstances [116]. In this sense, genomic and related technological tools could highlight the genes that mitigate the stress effect so that efforts are conducive to maintain those genes in successive breeding programs [117]. The molecular level of stress-tolerant genes is in cross talk quantitative loci traits showing their interaction and cloning of the genes that are related to stress [118]. In general, it is accepted that a combination of selection through marker assessment, molecular and traditional breeding as an integrated approach is the best alternative for the improvement of the abiotic stress tolerance in plants genetic engineering [119,120].

## 6. Drought-Resistance Induction

Plants adopt various approaches and strategies to alleviate the adverse effects of drought stress. Agriculturists are also using various strategies for drought stress tolerance, among which the application of exogenous regulators, chemicals, synthetic hormones and compounds are of great value to increase drought resistance at different plant growth stages.

### 6.1. Seed Priming

Seed priming has been referred to as the most important short-term approach to alleviate the adverse effect of drought on plants [126] (Table 2). The objective of this pre-sowing technique is to initiate the germination process in the metabolic machinery of the seed and prepare the seed for radicle protrusion without radicle emergence taking place during the process [127]. The germination process of prime seeds is more efficient, which results in higher germination rates and uniformity compared to non-primed seeds [128]. In crops like wheat, maize and chickpea seed priming is used to alleviate the adverse effect of drought stress [126,127,128]. Recently, the directly seeded rice (DSR) method used in rice grown in aerobic conditions resulted in an increment in the drought severity and frequency [129]. Under water scarce conditions, different osmotica were used for DSR with the result that CaHPO4 and KCL osmopriming enhanced crop yield and productivity. Better germination and stands were observed in primed seeds in water scant areas [128,130]. Optimal stand, better yield, ability to withstand drought, early and synchronized germination followed by emergence are linked with seed priming. It is reported that primed seed enhanced WUE by 44% in wheat crop than non-primed seeds under water stress conditions. High grain yield with early emergence and flowering resulted in primed seeds in water limited environments. Similarly, osmopriming with KNO3 and hydropriming enhanced yield of certain crops in water scarcity [131].

### 6.2. Plant Growth Regulators

Application of natural and synthetic plant growth regulators (Table 3) can improve drought tolerance in plants [142]. The reduction in the length and weight of the hypocotyl in seedlings due to water stress can be mitigated with the application of gibberellic acid (GA), which helps to maintain the internal water balance and the protein synthesis in drought stressed plants [143]. The stomatal conductance, as well as the photosynthesis and the respiration rates in wheat and cotton and maize were increased in water-scant areas following application of GA, and this resulted in higher grain yields compared to treatments where GA was not applied [143,144]. Exogenous application of abscisic acid, uniconazole, brassinolide and jasmonic acid can also improve crop productivity under drought [145,146]. Another active cytokinin, benzyladenine, is a hormone that regulates the drought resistance mechanism in various plants, including maize, wheat, cotton, chickpea barley and rice soluble sugar, soluble protein content, and the activities of superoxide dismutase, peroxidase, and catalase in the leaves were increased by uniconazole and brassinoloide in drought stress conditions [147].

Salicylic acid, an exogenously applied substance also improves drought tolerance and enhances growth and final harvest of the plants under water scarcity [148]. An enhancement in the catalase activity of wheat was observed through salicylic acid application under water-scarce conditions [149]. Use of salicylic acid and its derivatives in foliar and seed treatment applications increased the drought tolerance mechanism in wheat crop subjected to drought stress. Research shows that application of salicylic acid in wheat indirectly increased the accumulation of proline through an increment in the abscisic acid content [148,149]. In maize (*Zea mays* L.), polyamines contents are increased under drought stress conditions. Phytohormones such as ethylene and brassinolide (BR) are also of great importance to cope with various environmental stresses, especially drought stress. It enhances plant tolerance to biotic and abiotic stresses, through a complex pathway to regulate the plant defense system, by activating BZR1/BES1 transcription factors. It also regulates reactive oxygen species (ROS) production in plants under stress, and unbalancing of ROS scavenging leads to oxidative bursts, which have adverse effects on plants [150,151].

### 6.3. Osmoprotectants

The multiple range of plant stresses that reduce plant growth and productivity are regulated (Table 4) by osmoprotectants signaling. These substances accumulate during the time when growing conditions are not suitable for plant growth and development, and are responsible for maintaining the internal physiological processes that ensure plant survival under optimal conditions such as water scarcity [161,162]. Among others, important osmoprotectants in plants subjected to water stress include proline, trehalose, mannitol, fruton, and glycinebetaine [163]. These compounds, typically used for seed treatment or exogenously applied at different growth stages of established crops, protect the subcellular structure, increase the activity of antioxidant enzymes and mediate the osmotic adjustment in water-stressed plants [164,165]. Foliar application of proline also enhances the internal free proline in plants thereby increasing their drought tolerance [166]. Finally, use of polyamines like spermidine have also demonstrated to be efficient to increase plant tolerance to water stress in crops like barley (*Hordeum vulgare* L.) and wheat [167].

### 6.4. Silicon, An Abundant Element on Earth

The most abundant element on the earth surface, silicon, could be used as a mineral nutrient to increase plant resistance (Table 5) to various levels and degrees of stresses [173] and the overall mechanical strength of both stressed and non-stressed plants [174]. Moreover, exogenous application of silicon has demonstrated the capacity to increase the relative water contents in sorghum and sunflower [173,174]. Additionally, and compared to the unfertilized control, wheat plants applied with silicon not only maintained higher relative water contents but also increased the shoot dry matter when exposed to water stress conditions. Thus, silicon application decreased the shoot to root ratio through root growth facilitation. Furthermore, silicon application to wheat increased the photosynthesis rate, stomatal conductance and the antioxidant defense compared to plants with no silicon application [175]. Thus, silicon application in crops exposed to drought conditions can play an important role in maintaining the growth of roots and transport of water under drought stress [173,176].

### 6.5. Selenium As An Antioxidative Protectant

The plants exposed to water stress deficit produce ROS that can cause and oxidative damage to the biomolecules such as carbohydrates, proteins, lipids and nucleic acids; and therefore, reducing the photosynthesis, respiration and growth of plants [182]. Selenium (Se) application can result in compatible solutes in the plants grown under water deficit; and thereby reducing the oxidative stress in plants. The cellular dehydration of the plants is reduced through the accumulation of these osmolytes [183]. Senescence is stimulated in the plants as a result of an oxidative stress protection that produces the ROS enzymes under Se application to the plants [184,185]. Protective enzymatic activities are also activated through Se application in plants [186]. The application of Se enhances the production and synthesis of proline and peroxidase through antioxidant effect. Its application in plants can decrease the membrane degradability and enhance ROS enzymes activity [184,185,187]. Moreover, Se application can enhance plant growth, reduce oxidative stress damage, increases oxidative stress under light stress, antioxidants production due to senescence and regulating water balance of the plants to tolerate drought stress [188].

### 6.6. Potassium: A Vital Regulator

Potassium (K) application under drought stress condition ameliorates the adverse effect (Table 6) of the water deficit and maintains the plant productivity. Under drought stress condition, the plants uptake more potassium for their internal regulatory mechanism [189]. The increase of K by plants cause an oxidative damage, and therefore can form ROS during the photosynthesis process [190]. Thus, the reason of the high K demand by plants grown under stress is to maintain the CO_2_ fixation during photosynthesis process. Under plant stress, the increment of ROS in plants can be due to CO_2_ reduction [191]. The photosynthesis process was impaired, and carbohydrate metabolism was also affected through ROS production when plants were grown under water deficit conditions [192]. The low photosynthesis rate was observed in plants grown under drought stress with the lower dose of K application than higher dose of K [193]. Therefore, adequate K is needed for plants to maintain their physiological processes. It is also observed that the low grain yield of crops grown under water deficit condition could be enhanced through K application. The application of K as soil amendment or as foliar application is beneficial for the optimal physiological processes of plants [194,195]. Consequently, K application is of great importance for getting optimal yield production of crops grown under rained and/or water deficit environments [196].

### 6.7. Plant Microbes Crosstalk

**Figure 4:**
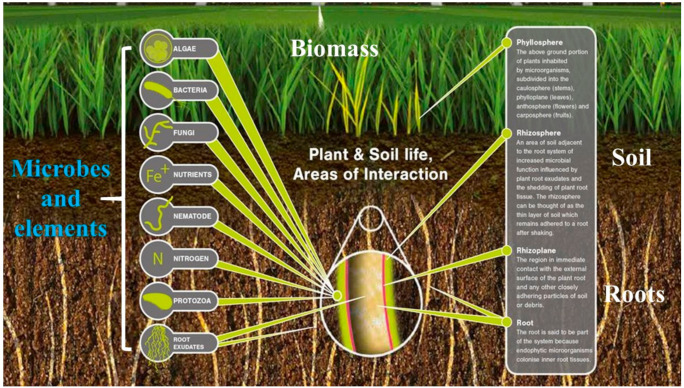
Schematic representation of the interaction among microbes, soil and plant, modified from Andreote et al. [213].

The microorganisms also play a vital role in reducing the adverse effects of drought stress (Table 7) and thereby improving plant productivity [202]. The oxidative damage in the plants grown under different environmental stresses can be reduced through the microorganisms (Figure 4) and enabling the cereals to cope with drought conditions. Among them, plant growth-promoting rhizobacteria (PGPR) is responsible for drought stress effect mitigation in dry environments [203]. The PGPR inoculation into the plants can increase the drought tolerance of those crops [204], because these PGPR make colonies in the root-zones and enhance the plant growth under different circumstances [205]. They also can solubilize various micronutrients to make them available for the plant uptake [202,206]. PGPR also enhances the plant resistance to different abiotic stresses [207]. The *Bacillus* species assembles solutes that enable maize plants to cope with drought and prevent degeneration [208]. In rice plants, the biotic and abiotic stresses were mitigated through phyllosphere bacteria inoculation [209]. The inoculation of *Bacillus amyloliquefaciens*, *Azospirillum brasilense*, *Rhizobium leguminosarum*, *Mesorhizobium cicero* bacterials strains improved homeostasis in plants and increased growth, biomass and drought tolerance index [210]. Similarly, *Trichoderma* sp. was reported to be a beneficial for drought stress [211], particularly *Trichoderma harizianum* was noted to be a beneficial application for rice drought tolerance [212].

### 6.8. Hydrogel: A Water Absorbing Polymer

Hydrogel is a polymer, and its application to the soil in agriculture systems can reduce the need for frequent irrigation [228]. Plants can survive and sustain their life cycle through hydrogel conditioning in arid and semi-arid environments, where the shortage of water is a serious issue [229]. The water limitation is not covered with the rainfall occurrence, and hence there is a demand to protect the available soil moisture from damage and loss to overcome soil degradation [230]. Due to hydrogel soil amendment, soil physical, chemical and biological traits are enhanced with positive effects on the plant growth and development [231]. Through its application to soil, it increases the plant survival time under drought stress, which was decreased due to the loss of the water and the hydraulic conductance in soil [232]. The survival time of the plants was increased with the hydrogel application since it resulted in sufficient soil moisture. Therefore, its application into the soil, particularly in arid and semi-arid environments and drought-affected areas, is beneficial for water saving in rhizosphere [233]. Apart from this, the hydraulic conductivity of the polymer amended soil is less than the plain soil. Similarly, water loss through evaporation in polymer-amended soil was lower than the soil with no hydrogel amendment [234].

### 6.9. Nanoparticles; Coping Drought Stress

Nanoparticles (NPs) are characterized by its particle shape, tunable pore size, potential reactivity and high surface area [235]. In plants, the cellular organelles are targeted, and certain contents are released through the nanoparticle target [235,236]. The activity of antioxidants enzymes i.e., SOD, CAT and POD were regulated and enhanced (Table 8) by the application of nanoparticles [237]. For example, the activity of SOD in plants was increased by the application of TIO2 NPs [238]. In agriculture, different trace elements and their oxides of NPs were used for enhancing drought stress resistance in different plants (Table 8). The negative effects of abiotic stress such as drought, chilling stress, salinity and heavy metal toxicity were mitigated through silicon nanoparticles (Si-NPs) application [235,239]. Growth and physio- and biochemical traits such as proline, chlorophyll, carbohydrates, carotenoids and relative water contents were significantly improved in different plant species when NPs were applied such as silica and ZnO nanoparticles [235]. Si-NPs also enhanced the drought resistance in wheat plants [240,241]. Similarly, the salinity and drought stress in plants were also mitigated by ZnO nanoparticles application (235). During the early stage of growth, the application of ZnO NPs stimulated the seed reservoirs for sapling and enhanced the drought resistance in plants [242]. Ferrous in combination with Zn were also reported to have a beneficial effect on plant resistance to drought stress. Plants grown under drought stress were mitigated through TIO_2_ nanoparticles, consequently activated different compounds and ameliorated the adverse effects of water deficit [243,244]. To improve drought stress in plants, other NPs such as silver (Ag) and copper (Cu) were used in lentil for mitigated drought stress negative effects. Nano-silica could also enhance the drought tolerance in different plants [235]. The increase of SOD and POD activity in wheat crop as drought resistance mechanism was observed through ZnO NPs. The drought resistance in wheat was also enhanced under Zn and Cu NPs [241,245].

### 6.10. Metabolic Engineering and Stress Tolerance Strategy

One of the most optimal solutions for coping drought stress is the drought tolerant crops development [252]. Thus, a great challenge is to enhance the drought tolerance without a significant effect on grain yield. The drought tolerance induction in plants through metabolic engineering, thereby enhancing stress related metabolites, is considered as an optimal strategy [253]. In arid and semi-arid regions, the successful breeding for drought tolerance through raffinose biosynthesis engineering pathway is one of the classic strategies. The accumulation of raffinose and galactinol in plants grown under water deficit is stimulated through galactinol synthase (AtGolS) gene with specific gene AtGolS2 that is stimulated under drought stress in particular [254]. The expression of this gene in plants enhances the raffinose and galactinol level, thereby enhancing drought tolerance in plants as well as protecting them from an oxidative stress. Both the raffinose and galactinol exhibits the potential to protect cell under environmental stresses through ROS scavenger and compatible solutes [254]. In this respect, the increment in raffinose and galactinol levels under metabolome analysis of rice and soybean indicated their response to drought stress. Crop plant transformation through AtGolS2 application activates the plants’ resistance to stress under dry environments. Different studies suggest that the application of AtGolS2 in transgenic plants not only increase drought tolerance but improves also grain yield [255]. Thus, AtGolS2 metabolic engineering is considered a useful approach and a significant tool to increase grain yield under water deficit conditions [256].

## 7. Conclusions

Under recent climatic changes, both the biotic and abiotic stresses are a serious threat for global food security and plant production sustainability. Among the abiotic stresses, drought stress is gaining attention due to its adverse effect on plant growth and development and significant reduction in plant yield and biomass causing global food insecurity. Drought stress affects plants through the life cycle i.e., from germination till maturity. Certain physiological, metabolic and biochemical processes are affected by drought stress that hampers plant productivity. To tackle the adverse effect of the drought stress on plants, certain mechanisms are adopted by the plants which enhance drought tolerance. Thus, there is need to explore the untapped adaptation characters in different plants and their incorporation to the genotypes that may tolerate the adverse effect of drought stress in order not to affect its productivity. Breeding technologies has greater potential for increasing plant performance and production under water deficit. Certain approaches are receiving greater attention for coping drought in arid and semi-arid environments.

Growth pattern and structural dynamics, reduction in transpiration loss through stomatal conductance altering and distribution, leaf rolling, root to shoot ratio dynamics, root length increment, accumulation of compatible solutes, enhancement in transpiration efficiency, osmotic and hormonal regulation and delayed senescence are the strategies that can be adopted by plants grown under water deficit.

To improve drought stress tolerance in plants, certain breeding strategies, molecular and genomics perspectives with special emphasis on the omics technology alteration i.e., metabolomics, proteomics, genomics, transcriptomics, glyomics and phenomics approaches are of great value. Other practices that include seed priming, growth hormones, osmoprotectants, silicon (Si), selenium (Se) and potassium application are worth using in scant water conditions in plants. Despites this, the beneficial effect of microbes, hydrogel, nanoparticles applications and metabolic engineering techniques also regulates the antioxidant enzymes activity for adaptation to drought stress in plants, enhancing plant tolerance through maintenance in cell homeostasis and ameliorates the adverse effects of water stress in plants. These innovative strategies provide better understanding of and potentially increase plant productivity in dry environments in order to reduce the threat to global food security.

## Figures and Tables

**Table 1:** Genes responsible for drought tolerance in plants.

Host Plant	Gene Responsible	Function	Reference
Wheat	TaNAC69	Increased tolerance to drought	[121]
Maize	NF-YB2	Under drought it enhances yield and photosynthetic rate	[122]
Rice	AP37, OSNAC10	Drought tolerance and grain yield increased	[123]
Soybean	P5C5	Improvement in drought tolerance	[124]
Sugarcane	SodEFF3	Drought tolerance increased	[125]
Tobacco	HSP70-1	Drought stress tolerance mechanisms enhanced	[120]

**Table 2:** Tolerance mechanisms in different field crops through seed-priming treatments.

Priming Method	Crop	Protective Effects	References
Cacl2 Hydro- and Osmopriming	Rice	Phenols, falovnoida accumulation, antioxidant system and enhances stand establishment	[132]
SNP Osmopriming		Compatible solutes accumulation enhances RWC, photosynthetic capacity, membrane stability and antioxidant enzymes	[133]
On-farm priming	Maize	Sustains optimal temperature for germination and less emergence time	[134]
Hydropriming	Canola	Growth of seedling, root shoot ratio and germination enhanced	[135]
Molecular priming	Plants	Induce tolerance against oxidative stress	[136]
KNO_3_ Priming	Soybean	Number of grains and pods per plant increased	[137]
Hydropriming	Cotton	Seed vigor and germination enhancement, thermal time reduction for emergence of radical	[138]
Bio- and Osmopriming		Increment in LA, phenols, clorophyll contents, grain yield and quality	[139]
Osmopriming	Sunflower	Catalase synthesis and immunocytolocalization increased	[138]
Osmopriming	Wheat	Maintains RWC, enhances proline accumulation, chlorophyll contents and emergence of leaf	[140]
CaCl_2_ Osmopriming		LPO reduction, osmolyte accumulation, increment in LA, RWC and grain yield	[141]

RWC: Relative water contents, LPO: Lipid peroxidation, LA: Leaf area, SNP: sodium nitroprusside.

**Table 3:** Tolerance mechanisms to drought enhancement through phytohormones in different field crops.

Host Plant	Phytohormone	Mechanism	Yield Dynamics	References
Potato	Auxin	ROS and water loss reduction	Increased 10%	[152]
	Jasmonic Acid (JA)	Root and shoot length increased, water loss decreased, plant defense and oxidative stress changed		[153]
Soybean	Abscisic Acid (ABA)	Stress genes regulated, proline and antioxidative enzyme activity increased and reduction in stomatal size	21% increment	[154]
Rice	Gibberellic Acid (GA)	Maximum WUE, photosynthesis, APX, CAT, proline contents, expanded roots, and dwarf plants	10–30% increased	[155]
Cotton	ABA	SOD, CAT, chlorophyll and proline increases	46% increase	[156]
Rice	ABA	Longer roots, reduced stomatal density, size and leaf area, while ABA, proline, soluble sugar and SOD increased	16% increased	[157]
Maize	ABA	Increased ABA accumulation and drought tolerance	Increased	[158]
Barley	Cytokinins	Transgenic barley plants showed better drought tolerance via better dehydration avoidance	Increased	[159]
Tomato	GA	Reduced whole-plant transpiration, smaller and reduced stomatalpores	Increased	[160]

Abbreviations: WUE: Water use efficiency, APX: Ascorbate peroxidase, CAT: Catalase, SOD; Superoxidase dismutase, ROS: Reactive oxygen species.

**Table 4:** Osmoprotectants significance in drought tolerance mechanisms different plants species.

Osmolytes	Plants	Plants Mechanism	References
Glycine betaine	Maize, Rice, Barley	Photosynthetic efficiency maintenance, thalakoid membrane protection and osmotic adjustment	[168]
GA& ABA	Tobacco	Improves stress tolerance, scavenging of ROS and carbon nitrogen balance	[169]
Fructan	Sugar Beet	ROS scavenger, protein and membrane stabilization and osmotic adjustment	[170]
Mannitol	Maize	Scavenge the stress induce oxygen radicals and osmotic adjustment	[171]
D-Ononitol	Arabidopsis	Prevent water loss in plants	[172]

ROS: Reactive oxygen species.

**Table 5:** Silicon activates the antioxidants activity and improves drought tolerance mechanisms plants.

Crop Plant	Activity	Reference
Tomato	CAT, SOD and GR activity increased	[177]
Tomato	Increment in CAT and SOD activity while reduction in POD activity	[178]
Wheat	CAT, SOD and GR activity increased	[179]
Sunflower	APX and MDA activity reduction	[180]
Wheat	Increment in ascorbate contents	[181]

Abbreviations: CAT: Catalase, SOD; Superoxidase dismutase, GR: Glutathione reductase, POD: Peroxidase, APX: Ascorbate peroxidase, MDA: Malondialdehyde.

**Table 6:** Potassium application mitigates the adverse effects in plants subjected to water deficit stress.

Plant Species	Water Stress Level and Time	Potassium Rate	Advantages	References
Wheat	PEG @ 15%	10 mM K_2_O	Proline contents, chlorophyll a, b and carotenoids increased	[197]
Sunflower	Withholding irrigation at grain filling	100 kg ha^−1^	shoot dry matter and biomass increased	[198]
Rice	30 DAP for 10 days	120 kg ha^−1^	shoot dry matter increased and osmolyte synthesis enhanced	[199]
Maize	65% of FC water holding	0.42 g kg^−1^ of soil	K^+^, glycine betaine and osmotic nitrides accumulation increased	[200]
Barley	50% of soil moisture	10 mM K_2_CO_3_	Reduction I soluble carbohydrate and enhanced K in plants	[201]

**Table 7:** Effect of microbes on plant adaptive mechanism for mitigation of drought stress.

Specie/Plant Name	Microbes	Activity	Ref
Maize	*Azospirillum lipoferum*	Increase accumulation of soluble sugar, free amino acids and proline. Affect the growth of root length, shoot fresh weight, shoot dry weight, root fresh weight and root dry	[214]
	*Bacillus spp.*	Increased accumulation of proline, sugars, free amino acids and decrease electrolyte leakage. It also reduce the activity of antioxidants enzyme (catalase, glutathione peroxidase)	[215]
*Helianthus annuus*	Pseudomonas putida strain GAP-P45	Epoxy polysaccharide production	[216]
*Capsicum annum*	*Bacillus licheformis* strain K11	Stress related genes and proteins	[217]
Rice	*Trichoderma harzianum*	promote root growth independent of water status and delay drought response	[218]
*Phaseolus vulgaris*	*Rhizobium tropici* and *Paenibacillus polymyxa*	Upregulation of genes involved in stress tolerance	[219]
*Medicago truncatula*	*Sinorhizobium medicae*	Root nodulation and nutrient acquisition of nutrient during drought stress	[220]
Wheat	*Bacillus amyloliquefaciens* 5113	Bacterial mediated plant attenuated transcript level and improves homeostasis	[221]
	Azospirillumbrasilense NO40		
*Brassica oxyrrhina*	*Pseudomonas libanensis* TR1 and *Pseudomonas reactans* Ph3R3	Increased plant growth, leaf relative water and pigment content and decreased concentrations of proline and malondialdehyde in leaves	[222]

*Cicer arietinum* L.	*Pseudomonas putida* MTCC5279 (RA)	Osmolyte accumulation, ROS scavenging ability and stress-responsive gene expressions	[223]
Lettuce	*Azospirillum sp.*	Promoted aerial biomass, chlorophyll and ascorbic acid content, as well as enhanced overall visual quality, hue, chroma and antioxidant capacity, and reduced the browning intensity	[224]
Arabidopsis	*Piriformospora indica*	Drought tolerance	[225]
Soybean	*Pseudomonas putida* H-2–3	Reduce the level of abscisic acid and salicylic acid and increase level of jasmonic acid content. Modulated antioxidants by declining superoxide dismutase, flavonoids and radical scavenging activity	[226]
Wheat	*Azospirillum brasilense* NO40	Catalase, exopolysaccharides and IAA produced by the Rhizobia improved the growth, biomass and drought tolerance index	[227]
	*Mesorhizobium ciceri* (CR-30 and CR39), and *Rhizobium phaseoli* (MR-2)		

**Table 8:** Drought stress tolerance enhancement in plants through Nanoparticles application.

Nanoparticles	Mechanism	References
Iron	Drought stress impacts on safflower yield components and oil percentage were mitigated through foliar spray of iron nanoparticles (Fe-NPs)	[246]
Silica	Si-NPs enhanced drought tolerance in plants	[247]
Titanium	Seed gluten and starch contents of wheat were improved through foliar application of titanium	[248]
Thiol-gated mesoporous silica	The encapsulated ABA release was controlled that enhances AtGALK2 gene thereby improved drought resistance in *Arabidopsis* seedlings	[249]
Zinc oxide	Germination rate and percentage of soybean were improved by the application of ZnO NPs	[250]
Zinc and copper	MDA accumulation was decreased with the increment in antioxidative enzymes and RWC under water deficit in the presence of Zn and Cu NPs applications	[251]
